# Selection of an Aptamer Antidote to the Anticoagulant Drug Bivalirudin

**DOI:** 10.1371/journal.pone.0057341

**Published:** 2013-03-06

**Authors:** Jennifer A. Martin, Parag Parekh, Youngmi Kim, Timothy E. Morey, Kwame Sefah, Nikolaus Gravenstein, Donn M. Dennis, Weihong Tan

**Affiliations:** 1 Department of Chemistry and Department of Physiology and Functional Genomics, Shands Cancer Center and Center for Research at the Bio/nano Interface, University of Florida, Gainesville, Florida, United States of America; 2 Department of Anesthesiology and Pharmacology, University of Florida, Gainesville, Florida, United States of America; University of Helsinki, Finland

## Abstract

Adverse drug reactions, including severe patient bleeding, may occur following the administration of anticoagulant drugs. Bivalirudin is a synthetic anticoagulant drug sometimes employed as a substitute for heparin, a commonly used anticoagulant that can cause a condition called heparin-induced thrombocytopenia (HIT). Although bivalrudin has the advantage of not causing HIT, a major concern is lack of an antidote for this drug. In contrast, medical professionals can quickly reverse the effects of heparin using protamine. This report details the selection of an aptamer to bivalirudin that functions as an antidote in buffer. This was accomplished by immobilizing the drug on a monolithic column to partition binding sequences from nonbinding sequences using a low-pressure chromatography system and salt gradient elution. The elution profile of binding sequences was compared to that of a blank column (no drug), and fractions with a chromatographic difference were analyzed via real-time PCR (polymerase chain reaction) and used for further selection. Sequences were identified by 454 sequencing and demonstrated low micromolar dissociation constants through fluorescence anisotropy after only two rounds of selection. One aptamer, JPB5, displayed a dose-dependent reduction of the clotting time in buffer, with a 20 µM aptamer achieving a nearly complete antidote effect. This work is expected to result in a superior safety profile for bivalirudin, resulting in enhanced patient care.

## Introduction

Anticoagulant drugs have some of the highest instances of adverse reactions and medication errors of all drug classes [1]. These actions directly correlate to an increased occurrence of complications, such as severe bleeding, that increase patient morbidity and mortality [2]. Blood transfusions are required for 5–10% of patients with severe bleeding, at an estimated cost of $8,000–$12,000 per incident [3]. In addition to cost, the negative effects of blood transfusion include anaphylaxis, immune suppression, poorer outcomes in cancer patients, infection (e.g., hepatitis), and others. Consequently, the selection of an anticoagulant drug must be carefully considered with a view towards possible safety issues. Ideally, a safe and efficacious antidote should also be available to reverse the effects of the anticoagulant and prevent or treat severe patient bleeding.

Heparin and protamine are the most well-known anticoagulant/antidote pair commonly used in clinics, but both drugs have considerable risk associated with their use. Heparin cannot inhibit fibrin-bound thrombin, possibly due to steric constraints. If heparin docks to thrombin without previously binding antithrombin, it can form a bond with thrombin-bound fibrin, actually strengthening the clot [4]. Heparin also binds to certain plasma proteins in the blood, resulting in an unpredictable anticoagulant response requiring increased patient monitoring. Also, heparin is neutralized by platelet factor 4 (PF4), a product of activated platelets [5]. Complexation of heparin with PF4 or other plasma proteins constitutes a major challenge in heparin use because it can stimulate heparin-induced thrombocytopenia (HIT), which can cause severe reactions in some patients. Approximately 600,000 (5%) patients out of an annual total of 12 million receiving heparin develop HIT and can no longer continue heparin administration [6]. Protamine, the antidote to heparin, also has serious side effects associated with administration, including increased and potentially fatal pulmonary artery pressure, decreased systolic and diastolic blood pressure, impaired myocardial oxygen consumption, and reduced cardiac output, heart rate, and systemic vascular resistance [2].

A variety of synthetic anticoagulant drugs has been developed to avoid the challenges posed by heparin use. In particular, bivalirudin is a 2180 Da synthetic peptide anticoagulant that has several advantages over heparin. Bivalirudin generates a more predictable anticoagulant response because it does not bind to other plasma proteins. It also binds both fibrin-bound and free thrombin, is not inactivated in the presence of PF4, and does not induce HIT [4], [7]. Despite the advantages of using bivalirudin, the overshadowing drawback is that it currently does not have an available antidote. Therefore, the objective of this work was to provide an antidote to bivalirudin to introduce a safe and reliable anticoagulant/antidote pair.

To accomplish this, we implemented a method known as SELEX (Systematic Evolution of Ligands by EXponential enrichment) to select an aptamer antidote to bivalirudin. Aptamers are single-stranded DNA or RNA molecules selected to bind to a target molecule based on the specific three-dimensional conformation adopted [8]. The SELEX procedure begins with 10^13^–10^15^ unique sequences from a chemically synthesized, randomized oligonucleotide library. These sequences are then incubated with the target species, in this case, bivalirudin. Nonbinding sequences are partitioned from binding oligonucleotides, which are then eluted from the target. This partitioning is the main determinant of the efficiency of the selection. A counter-selection step may be included to remove sequences that bind to a predefined control or support matrix. The sequences which do not bind to the control are then amplified by polymerase chain reaction (PCR) and converted to single-stranded DNA (ssDNA) for the next round of selection. The process is repeated until the pool is enriched for sequences binding specifically to the target. A typical selection requires an average of 12 cycles, depending on the selection strategy and efficiency of partition, and a timeline of 2–3 months [9], [10].

Aptamers have shown particular promise in the role of anticoagulants, targeting various points of the coagulation cascade. Aptamers to thrombin [11], factor IX [12], factor VII [13], factor X [14], protein C [15], and von Willebrand factor [16] have been shown to successfully modulate thrombus formation, with complementary DNA (cDNA) antidotes able to restore normal activity. Specifically, Rusconi and coworkers developed the factor IX aptamer that is entering clinical consideration and testing. [12]. Upon addition of cDNA corresponding to the contiguous stem region at the 5′-end of the aptamer, the anticoagulant effect was neutralized and clotting activity commenced in animal models.

Because aptamer/cDNA development is relatively cost-prohibitive, studies have been carried out to find different methods to reverse anticoagulant binding [2]. Recent work has investigated the use of light to photoregulate aptamer antidote activity by introducing a “caged” structure into an aptamer [17] or an azobenzene moiety to reverse anticoagulation [18]. These designs may have topical applications, but they are limited by depth of UV/Vis penetration into the skin. Researchers have also used aptamers and cationic porphyrins to serve as anticoagulant/antidote pairs; however, the method functions only with aptamers known to form a G-quartet [19]. Additionally, a polymeric antidote has shown a promising universal antidote response for various anticoagulant aptamers, but the mechanism of action is still unclear, and the research is still in the preliminary phase [2]. The present work reports the first successful selection of an aptamer antidote for a currently existing pharmaceutical anticoagulant drug.

In this work, the anticoagulant drug bivalirudin was immobilized on a monolithic column to partition nonbinding sequences from binding sequences. Two rounds of SELEX performed with target-binding sequences facilitated generation of multiple aptamer candidates after 454 sequencing. The candidates were assayed for binding to the target bivalirudin by flow cytometry, and aptamers that demonstrated binding were characterized in terms of their affinity for the target molecule by fluorescence anisotropy (FA) assay. One of the aptamers was then tested for antidote activity in a buffer model, establishing a dose-dependent decrease in clotting time as the aptamer concentration was increased.

## Results and Discussion

### Drug Immobilization on Monolithic Disk

The binding sequences were partitioned from the nonbinding sequences by covalently linking bivalirudin to a column support matrix. Affinity columns have played an important role in generating aptamers for various small molecule targets since the initial SELEX study by Ellington and Szostak was conducted to select aptamers for small organic dyes [8]. The advent of monolithic columns has introduced new technology with inherent properties that may provide unique advantages for affinity-based column aptamer selection. Monolithic columns are essentially constructed from one continuous material in which the area interacting with the analyte is mainly on the surface instead of located in small pores. This allows for mass transport by convection on the surface of the material, rather than the diffusion-limited separations of traditional bead-based methods. This leads to a decrease in the pressure drop across the disk, allowing for faster flow rates and the use of simple peristaltic pumps, such as those found in low-pressure chromatography (LPC) instruments [20]. Moreover, the hydrophobic backbone of the typically polymeric-based matrices has also been reported to have low nonspecific adsorption [21], and the target molecule can be covalently attached to the column by a variety of chemistries, minimizing instances of target leaching.

The drug was immobilized on an epoxy-functionalized disk with a methacrylate (poly(glycidyl methacrylate-*co*-ethylene dimethacrylate)) backbone (see Figure S1). This disk was determined to have 1 mg of drug immobilized via UV/Vis absorption of the drug solution. For comparison to the drug-immobilized disk, a blank disk, consisting of only the disk support matrix, was also prepared.

### Method Development and Selection

**Figure 1:**
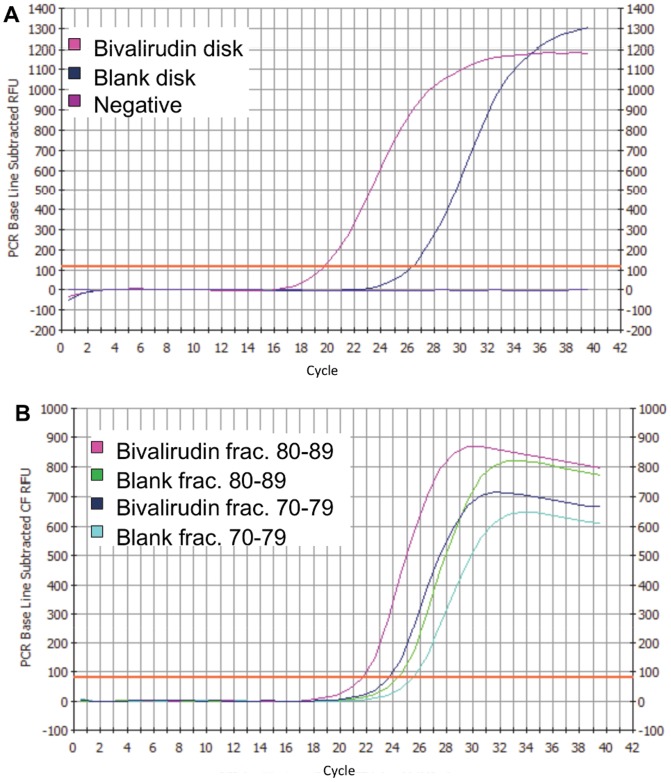
Real-time PCR analysis of fractions from drug and blank disk. A) Round 1 analysis; B) Round 2 analysis.

The gradient elution of binding DNA sequences was loosely based on the work of Giovannini and Freitag [22]. However, the return of the program to lower salt concentration was slowed in order to spread out the peak corresponding to binding sequences for collection into more fractions (Table S1). In the first round, 1 nmole DNA was incubated with both the drug-immobilized and blank disks. Fraction 15 from both the drug and the blank disks (see Figure S2) was compared via qPCR (real-time PCR). The drug disk contained more DNA (Figure 1A), as evidenced by the lower number of cycles required to reach the calculated qPCR threshold (19.64 cycles for the drug disk and 26.22 cycles for the blank disk). Because more DNA was present in the drug disk fraction than in the blank, it was assumed that some sequences in the drug fraction bound specifically to the target drug instead of solely to the support matrix. Therefore, all fractions contained in the chromatographic peak for the drug disk were combined and incubated with the blank disk to subtract out any sequences binding to the support matrix. The sequences which did not bind the blank disk were then PCR-amplified for the second round of selection (PCR amplification protocol shown in Figure S3).

In round 2 of selection, the amount of starting DNA was decreased to 200 pmoles for each disk. Collected fractions were pooled and analyzed by qPCR, with two of the pools (fractions 70–79 and 80–89) demonstrating a lower cycle number for the drug disk (Figure 1B). These pools were combined, PCR-amplified with FAM-labeled sense primers, and analyzed by AlphaScreen assay.

### Selection of Pool for Sequencing

An AlphaScreen (amplified luminescent proximity homogeneous assay) was used to determine the selected pool with the highest-affinity candidates for DNA sequencing. The method is a bead-based non-radioactive proximity assay that measures ligand/target interactions by the output of fluorescence of binding pairs [23]. In the assay, biotinylated target is immobilized on a streptavidin-coated donor bead that generates singlet oxygen upon excitation at 680 nm. This singlet oxygen initiates fluorescence (520–620 nm) in anti-FAM-coated acceptor beads that have FAM-labeled DNA pools immobilized on the surface. The fluorescence occurs only if the beads are within the defined distance (200 nm) that singlet oxygen can travel during the excited state lifetime. Therefore, the beads will generate measurable signal only if the ligand and target are bound, bringing the beads sufficiently close for the singlet oxygen to reach the acceptor bead.

**Figure 2:**
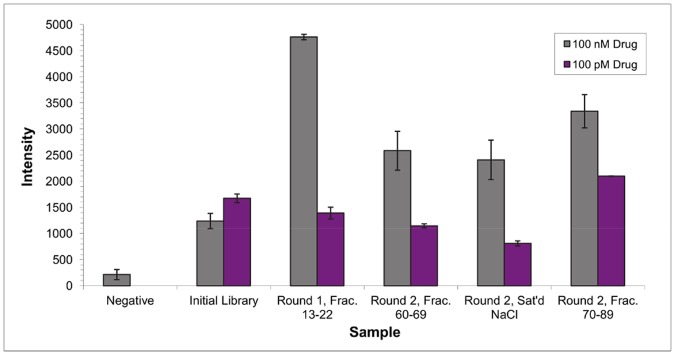
AlphaScreen analysis of selection pools.

The AlphaScreen assay was carried out at drug concentrations of 100 nM and 100 pM. The DNA obtained from the saturated NaCl soak after the elution protocol produced minimal signal at both drug concentrations (Figure 2). Round 1 displayed the highest intensity at high drug concentration, followed by round 2 fractions 70–89. However, the round 2 fractions 70–89 sample had the highest signal with the 100 pM drug concentration. These results suggested that the fraction from round 2 contained higher affinity sequences, because that fraction generated significant signal at high drug concentrations, as well as the highest signal at the lower drug concentration. Therefore, this pool was selected for 454 sequencing (preparation described in Text S1 and Tables S2 and S3) to yield ∼10,000 aptamer candidates.

### DNA Sequencing Analysis

Following 454 sequencing, the individual DNA sequences were analyzed using the MAFFT sequence alignment program. Several regions of homology were observed, even though only two rounds of selection were performed (example of JPB2 alignment shown in Figure S4). Seven of these aptamer candidates, which are listed in Table 1, were synthesized with a 5′-FAM fluorescent label for further characterization.

**Table 1:** Probe Sequences.

Name	Sequence
JPB1	ATC GTC TGC TCC GTC CAA TAC GAG GAT GCA GAA GTT TCA ATG CAC TTT TGG TGT GAG GTC GTG C
JPB2	ATC GTC TGC TCC GTC CAA TAC GTA ACA TCC CCG TAA TAC TAC TAC GGT CGT GCT GGT TTG GTG TGA GGT CGT GC
JPB3	ATC GTC TGC TCC GTC CAA TAG CTG AGC AGG TAA CAA TGT GTG CCC AAT GTG TAT TTG GTG TGA GGT CGT GC
JPB4	ATC GTC TGC TCC GTC CAA TAA AGT TAA TCC TTA GGG CTG GTA GGT CAT TCC GGT GGT TAT TTG GTG TGA GGT CGT GC
JPB5	ATC GTC TGC TCC GTC CAA TAT ATT GTG TGA CCC CCC TCT TGT TTT GGT GTG AGG TCG TGC
JPB6	ATC GTC TGC TCC GTC CAA TAC CAG CTA ATG TGT ATT TTG TGG CGG CGG ATC ATA TGA GGA GGA TTT TTG GTG TGA GGT CGT GC
JPB7	ATC GTC TGC TCC GTC CAA TAG TCG GAA TAG TGA CTG TTC TTG TGA AAC TCA ACA CGG ATG CTG GTG TTT TGG TGT GAG GTC GTG C

### Flow Cytometry Binding of Aptamer Candidates

**Figure 3:**
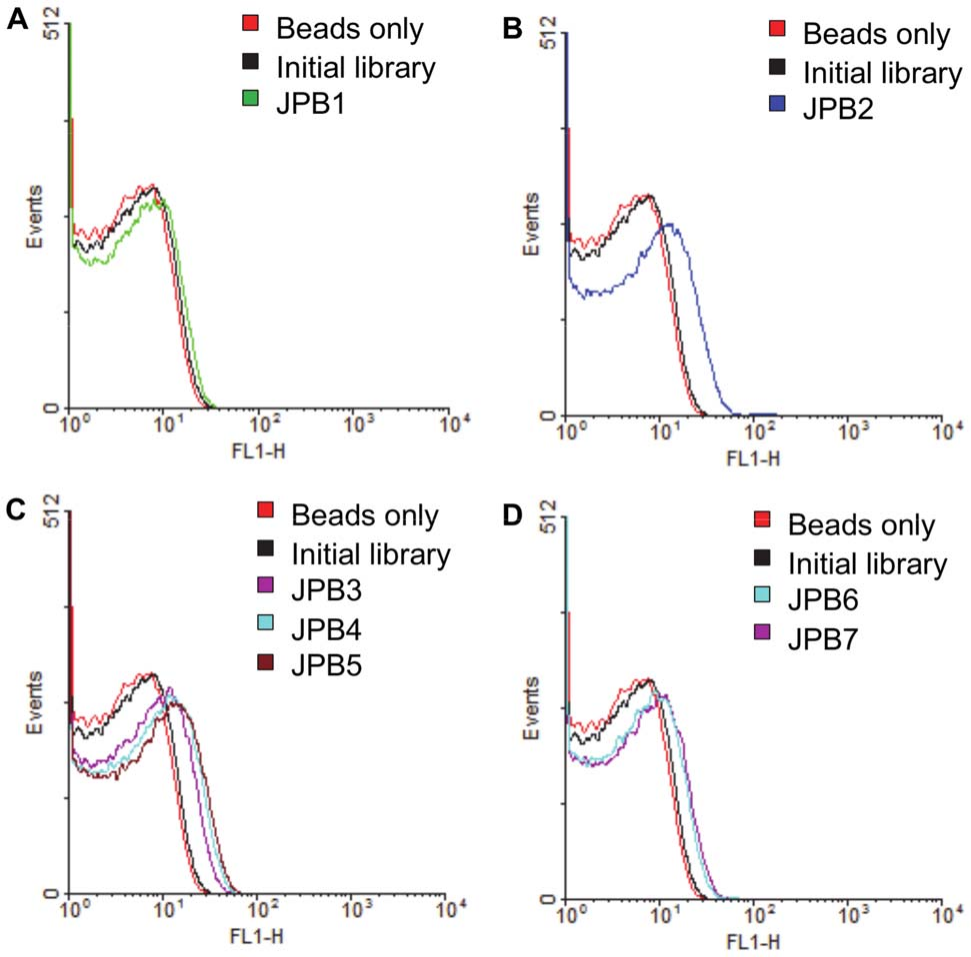
Flow cytometry binding studies of drug with aptamer candidates. A) JPB1; B) JPB2; C) JPB3–5; D) JPB6–7.

The assay to determine whether the sequences are binding the target is the critical stage of post-SELEX oligonucleotide characterization; sequences that bind are designated “aptamers.” Binding studies were performed by flow cytometry on all sequences listed in Table 1. Figure 3 shows that all of the sequences, except JPB1, demonstrated a significant increase in fluorescence intensity upon binding to the drug. Therefore, six aptamer sequences were successfully obtained as a result of this selection. The largest fluorescence intensity shifts were demonstrated by JPB2 and JPB5, and binding affinities were determined for these two candidates.

### Fluorescence Anisotropy for Dissociation Constant (K_d_) Measurements

Fluorescence anisotropy (FA) was utilized to determine the dissociation constants for JPB2 and JPB5. One distinct benefit of FA is that the method can be carried out in free solution, whereas methods such as surface plasmon resonance (SPR) or flow cytometry require the immobilization of the target or aptamer prior to measurement. In FA, the lower molecular weight component (drug) is typically labeled with the fluorescent reporter (described in Text S1) to maximize the change in anisotropy when the higher molecular weight component is titrated into the system.

**Figure 4:**
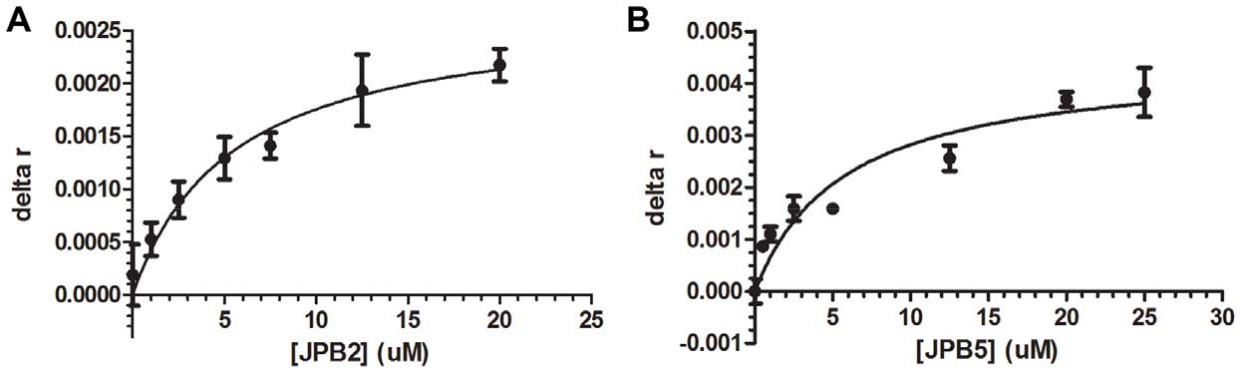
Dissociation constant measurements by fluorescence anisotropy. A) JPB2; B) JPB5.

In this work, the mass of bivalirudin (labeled with TMR dye) was kept constant, while increasing amounts of aptamer were titrated into the sample. Figure 4 demonstrates the change in anisotropy versus aptamer concentration using a single-site ligand binding model. The results show dissociation constants in the low micromolar range for both probes tested. The K_d_ of JPB2 was 5.99±0.88 µM, while that of JPB5 was 5.76±1.79 µM. These K_d_’s are on the high-affinity end of the spectrum of those reported for small molecules/peptides, with dissociation constants typically in the high nanomolar to high micromolar range [24], [25], [26]. As a control, the sequence TV03, previously selected for virus infected cells, was analyzed by FA using bivalirudin as a target [27]. The curve generated by TV03 was fit to the same binding model using GraphPad Prism, and a K_d_ estimate of 502.1 µM was generated (Figure S5). (Note that GraphPad is able to fit data and estimate K_d_ as long as the concentration range tested generates a nonlinear curve, although the error reported on the K_d_ is significantly higher when saturation is not obtained. Thus, the control TV03 curve is provided to compare the shapes of the binding curves of the aptamers to a nonbinding sequence in a similar concentration range.) This higher dissociation estimate across a similar concentration range shows that the dissociation constants determined for JPB2 and JPB5 are the consequence of specific binding of the sequences selected for the target molecule bivalirudin.

The K_d_’s reported for the aptamer/bivalirudin binding are higher than those of the drug with thrombin, but this does not mean the aptamers will not function as an antidote. Two modes of operation are possible for antidote binding, i.e., the aptamer may compete with bound thrombin for drug binding, which releases the drug from the complex and, as a result, restores coagulation, and/or the aptamer may inhibit free drug from interacting with thrombin [28]. Addition of an excess of aptamer to the system can help to increase the aptamer/drug interaction.

### Clotting Studies in Buffer

A simple clotting experiment was designed in buffer to determine the ability of JPB5 to restore coagulation activity to the system. The underlying principle is that thrombin will convert soluble fibrinogen to insoluble fibrin, which can then be monitored as a function of increasing light scattering of the solution [18]. The time required for fibrin conversion was compared for three different metrics. The normal clotting time, which denotes the time for thrombin to convert fibrinogen to fibrin devoid of the influence of the drug or aptamer, is expected to display a relatively short time and serve as a control value. The prolonged clotting time measures the effect of bivalirudin on the system, which will inhibit thrombin and increase the coagulation time (a positive control). Finally, various concentrations of aptamer are added to a mixture of thrombin and bivalirudin. If the aptamer is able to bind the anticoagulant and inhibit bivalirudin activity, a progressive decrease in coagulation time will be observed when higher JPB5 concentrations are administered.

**Figure 5:**
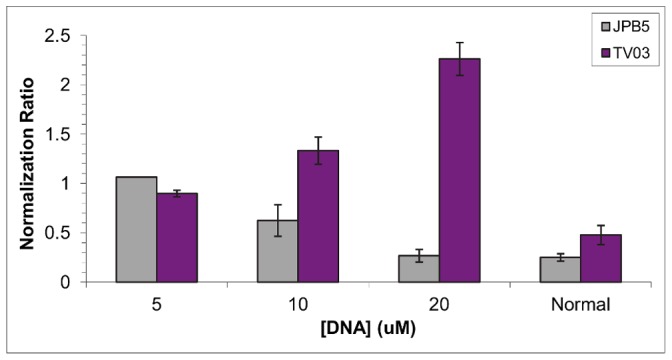
Clotting studies with JPB5 and TV03 control.

The results of the studies, performed on both JPB5 and a non-binding control sequence, TV03, are shown in Figure 5 (optimization of conditions described in Text S1 and Figure S6). The clotting times for each concentration of JPB5 were normalized relative to the prolonged clotting time. Therefore, a normalization ratio below 1 indicates that the clotting time is shorter than that of the prolonged clotting time, while a ratio above 1 signifies a clotting time greater than that of the prolonged clotting time. The addition of JPB5 to the drug/thrombin solution decreased the clotting time in a concentration-dependent manner, with a concentration of 20 µM JPB5 returning the coagulation time of the system to approximately normal levels. In contrast, the control sequence TV03 actually increased the clotting time of the system. This may have been caused by dilution of the procoagulant factors in the experimental system. Since TV03 does not bind to the drug, the addition of the sequence in buffer only served to dilute the concentrations of enzyme and substrate in the solution, decreasing the catalytic rate of fibrinogen cleavage. Note that the normal and prolonged clotting times typically varied from run-to-run by a few seconds. This explains why the normalization ratio for the “normal” clotting time varies within the two runs. Overall, these results prove that the antidote function of JPB5 is a consequence of the aptamer binding specifically to the target rather than nonspecific DNA interactions.

### Conclusions

In only two SELEX rounds, we were able to select an aptamer to the anticoagulant drug bivalirudin. This aptamer was shown to act as an antidote to the drug in buffer. Aptamer JPB5, identified through next-generation 454 sequencing, demonstrates a dose-dependent decrease in clotting time, with a 20 µM aptamer concentration nearly completely restoring the function of the system back to the normal clotting time. Overall, this work reports the first aptamer antidote selected for a currently existing drug using a novel monolithic-SELEX method in fewer rounds than required in a conventional selection.

The ability of the reported method to select aptamers to a target in only two rounds deserves further discussion. Typically, researchers determine when to terminate a selection by monitoring the enrichment of the pool through the progression of the selection- that is, what percentage of the initial amount of DNA is actually binding the target. Once the binding amount plateaus, the pool is deemed “enriched” for the target, and is prepared for sequencing. The selection conditions are modified to be increasingly stringent throughout SELEX to facilitate this process and generate tighter binders.

One aspect that aided this method was the use of the monolithic column with the fraction collector as a way to group the eluted sequences in terms of the more stringent binding conditions imparted throughout the course of a single round (tighter binders will elute later). These grouped fractions were then assessed by qPCR, and the groups that demonstrated higher affinity for the target than the matrix were further evaluated by AlphaScreen. AlphaScreen essentially served to monitor selection enrichment, as round 2- fraction 70–89 signal was the only fractional group significantly higher than the initial library at both drug concentrations. The fraction was sequenced under the hypothesis that next-gen sequencing would require fewer rounds of selection by the higher percentage of sequences detected (compared to cloning) obviating the need for a highly evolved pool.

The application of 454 sequencing is viewed as crucial to the success of this project. In a traditional cloning/chain termination sequencing protocol, the pool to be sequenced is cloned into a vector prior to sequencing [29]. Because of low efficiencies associated with the cloning process, the pool must be highly evolved to ensure representation in the sequencing data. Also, chain termination sequencing typically generates only several hundred sequences, depending on the cloning conditions, compared to several thousand sequences reported by 454 sequencing. Therefore, a higher percentage of sequences present in the final pool are detected, and the large number of sequences reported allows for a higher likelihood of homologous sequences after alignment. It is possible that 454 sequencing results in fewer rounds of selection by the higher percentage of sequences detected, thus obviating the need for a highly evolved pool.

Overall, the keys to success in only two rounds were an increased partitioning of binders from nonbinders by the monolithic method enabling increased stringency within the course of each round of selection, followed by 454 sequencing allowing identification of binders from a less-enriched pool than conventional cloning.

Because cost-effective aptamer therapeutics are considered to have 40 nucleotides or fewer, work is currently underway to truncate the selected aptamer sequences [30]. We are also in the process of introducing modifications to the selected aptamers to increase *in vivo* stability of the sequences. Various base modifications, such as addition of polyethylene glycol end caps or introduction of locked nucleic acids (LNA) into the structure, have been shown to inhibit nuclease activity [24], [31]. Development of these aptamers into functional therapeutics will improve the safety profile of the anticoagulant bivalirudin, which has several advantages and is sometimes essential for use as an alternative to heparin. This work also provides a foundation for selecting aptamer antidotes for other pharmaceutical drugs and biological toxins (e.g., reptilian envenomation) which do not currently have an antidote available.

## Materials and Methods

### Buffers

Binding buffer (BB): PBS buffer (no MgCl_2_ or CaCl_2_) with 5 mM MgCl_2_; Elution buffer (EB): BB with 1 M NaCl; Washing buffer (WB): 4.5 g/L glucose and 5 mM MgCl_2_ in Dulbecco’s PBS with CaCl_2_ (Sigma).

### DNA Synthesis

DNA was synthesized on an ABI 3400 DNA/RNA synthesizer (Applied Biosystems) at the 1 µmole scale using a standard synthesis protocol (Text S1).

### Drug Immobilization

The drug was immobilized on an epoxy-functionalized monolithic disk (BIA Separations) according to the manufacturer’s instructions. Details of drug immobilization are described in Text S1.

### SELEX Conditions

The drug-immobilized disk or blank (no drug) disk was washed for 5 minutes with EB, then BB, both at 2 mL/min. Two milliliters of 500 nM DNA library (1 nmole) in BB was heated to 95°C for 5 min and snap cooled on ice. This DNA was incubated with the disk in a small dish for 30 min. The disk was then placed in the provided column housing and connected to the LPC device with an automatic fraction collector (Bio-Rad BioLogic BioFrac Fraction Collector). An elution protocol was designed, as described in Table S1, with a flow rate of 2.0 mL/min for each step. DNA elution from the disk was monitored by UV detection at λ_abs_ = 254 nm, and each fraction collected contained ∼680 µL of fluid. The same procedure was followed for the blank disk, with a separate aliquot of DNA library. Fractions were analyzed by qPCR (see method below), and those corresponding to the peak of the drug disk were combined and incubated with the blank disk for 30 min in a small dish. Sequences not binding to the blank disk were amplified by PCR, and amplification was confirmed on 3% agarose gel (Fisher) in Tris-Borate-EDTA (TBE) buffer (Fisher). The selected sense DNA strands were separated from the biotinylated antisense DNA by alkaline denaturation and affinity purification with streptavidin-coated Sepharose beads (GE Healthcare) to produce single-stranded DNA (ssDNA) for the second round of selection. Each disk was rinsed and soaked in a saturated NaCl solution to remove any remaining bound sequences prior to the next round. A similar protocol was carried out for the second round of selection, except that 200 pmoles DNA was used for incubation with each disk.

### PCR Conditions

All PCR mixtures (Takara) contained 50 mM KCl, 10 mM Tris HCl (pH 8.3), 1.5 mM MgCl_2_, dNTPs (2.5 mM), 0.5 µM each primer, and hot-start *Taq* DNA polymerase (5 units/µL). Conditions were: 95.0°C for 2.5 min, repeated cycles (30 sec each) of 95.0°C, 55.0°C, and 72.0°C, followed by 5 min at 72.0°C and an indefinite hold at 4.0°C, performed on a Bio-Rad iCycler. The PCR protocol is shown in Figure S3.

### qPCR Analysis

qPCR was carried out on a Bio-Rad iCycler with MyiQ software and detection instrumentation. Centricon centrifugal filters (Millipore, YM-10) were used to desalt the fractions prior to qPCR. PCR mixtures contained iQ SYBR Green Supermix with iTaq DNA polymerase (Bio-Rad) and 0.5 µM of each primer. The conditions were: 95.0°C for 3.0 min, 40 repeated cycles (30 sec) of 94.0°C, 55.0°C (30 sec), and 72.0°C (15 sec), followed by the final steps of 5 min at 72.0°C, then an indefinite hold at 4.0°C. All thresholds were auto-calculated by the MyiQ program.

### AlphaScreen Assay

DNA aptamer candidate pools were converted to ssDNA (see above method) after PCR amplification with the primer 5′-FAM-ATC GTC TGC TCC GTC CAA TA (IDT) to provide an immobilization method for AlphaScreen anti-FAM donor beads (Perkin-Elmer). Anti-FAM beads (20 µg/mL, final) and dye-labeled DNA pools (400 nM, final) were incubated for 1.5 hours in an OptiPlate 384-well microplate (Perkin-Elmer) under subdued lighting. Streptavidin-coated AlphaScreen donor beads (20 µg/mL, final; Perkin-Elmer) and biotinylated drug (100 nM and 100 pM, final) were then added to each well and incubated for 1.5 hours. The final volume in each well was 25 µL, and the buffer for this experiment consisted of BB with 50 mg/mL BSA to inhibit nonspecific binding. The positive control was 400 nM biotinylated FAM, substituted for DNA in the method described. Data were read in replicate on an EnVision microplate reader (Perkin-Elmer).

### Sequencing of Selected Pool and DNA Alignment

A 400 µL (total volume) 25 cycle PCR (Table S2) was performed on the pool selected by AlphaScreen assay. This dsDNA-amplified pool was prepared for sequencing by high-fidelity (Roche Applied Science FastStart High Fidelity PCR System) PCR amplification with fusion primers specific for 454 sequencing technology. The primer sequences used for the PCR were composed of a combination of the 454 fusion tag and the regular sense and anti-sense primers previously used for amplification (Text S1 and Table S3). The dsDNA product was purified by a QiaQuick PCR Purification Kit (Qiagen) according to manufacturer’s instructions. Sequencing of the selected pool was performed by 454 sequencing at the University of Florida ICBR. The sequences were aligned based on homology using MAFFT software to yield several aptamer candidates.

### Binding Studies

Streptavidin-coated beads (100 µL; Bangs Laboratories) were initially washed with 1 mL BB. The supernatant was discarded, and the remaining beads were resuspended in 1 mL BB. One µL of 10 µM biotinylated-bivalirudin was combined with 10 µL of streptavidin-coated polystyrene beads (Bangs Labs) and incubated for 1 hour on a shaker. The beads were centrifuged and reconstituted in 1 mL BB. The beads were split into 20 samples, and each aptamer candidate was added (final concentration of 2.3 µM) and incubated with the bivalirudin/bead complex for 1 hour. Beads were centrifuged, washed with 1 mL of BB, and then reconstituted in 200 µL WB for flow cytometry. The fluorescence intensity of the FAM-labeled sequences was measured with a FACScan flow cytometer by counting 20,000 events (Becton Dickinson) and analyzed using WinMDI.

### Fluorescence Anisotropy Measurement

FA measurements were performed using a Fluoromax-4 spectrofluorometer (Horiba Jobin Yvon) and a TMR-labeled drug. Details are described in Text S1.

### Clotting Studies

Utilizing a Fluoromax-4 spectrofluorometer, the settings for this experiment were as follows: λ = 500 nm; slit width = 3 nm; integration time = 0.1 sec; interval = 0.5 sec, temperature = 37°C. The normal clotting time was determined by adding 50 µL of 15.5 nM thrombin in BB to the cuvette and then equilibrating it in the instrument for 2 minutes (step 1). Next, 50 µL of 1 µM fibrinogen (500 nM final) was added and mixed well by pipette, and the increase in light scattering was monitored using FluorEssence software (step 2). The prolonged clotting time was determined by addition of 1.08 µL of drug (10.8 nM final) to step 1, followed by fibrinogen addition. For the aptamer studies, drug and 5–20 µM of aptamer or control sequence (TV03) were added in step 1, followed by fibrinogen addition. Each aptamer concentration was tested in duplicate, and each curve of light intensity versus time was fit to a 4-parameter sigmoid equation using GraphPad Prism to determine the clotting time.

## Supporting Information

Figure S1 **Immobilization scheme of bivalirudin to the monolithic column.**
(TIF)

Figure S2 **Chromatograms of selection rounds.** A) Round 1, drug disk; B) Round 1, blank disk; C) Round 2, drug disk; D) Round 2, blank disk. The green circles correspond to individual fractions collected.
(TIF)

Figure S3 **PCR protocol for amplification.**
(TIF)

Figure S4 **Sample alignment of JPB2 (green highlighted portion) using MAFFT.**
(TIF)

Figure S5 **Dissociation constant curve of control sequence TV03.**
(TIF)

Figure S6 **Optimization of conditions for buffer clotting experiments.** A) Optimization of fibrinogen concentration; B) Optimization of bivalirudin concentration.
(TIF)

Table S1 **SELEX DNA elution method**
(JPG)

Table S2 **PCR Preparation**
(JPG)

Table S3 **Fusion primer PCR**
(JPG)

Text S1 **Supporting Information**
(DOCX)
