# Supplementary figures and images for: Selection of an Aptamer Antidote to the Anticoagulant Drug Bivalirudin
Source: PLoS One. 2013 Mar 6;8(3):e57341. doi: 10.1371/journal.pone.0057341 (PMC3590194; doi:10.1371/journal.pone.0057341)

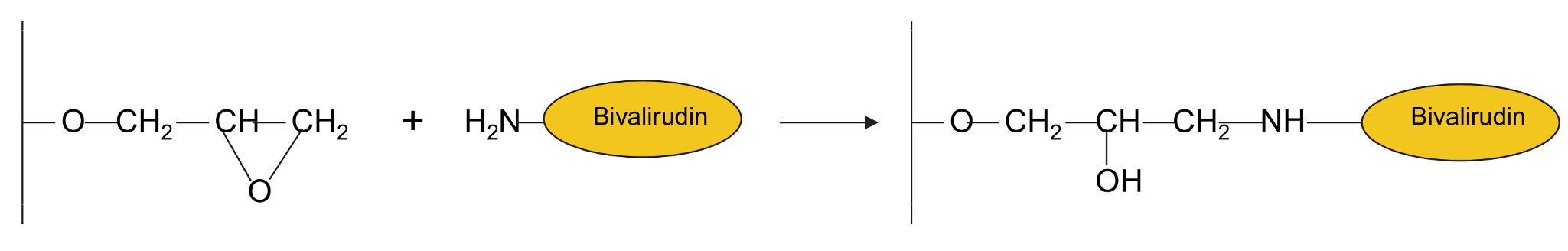

Supplement: Figure S1 — Immobilization scheme of bivalirudin to the monolithic column. (TIF) [file pone.0057341.s001.tif]

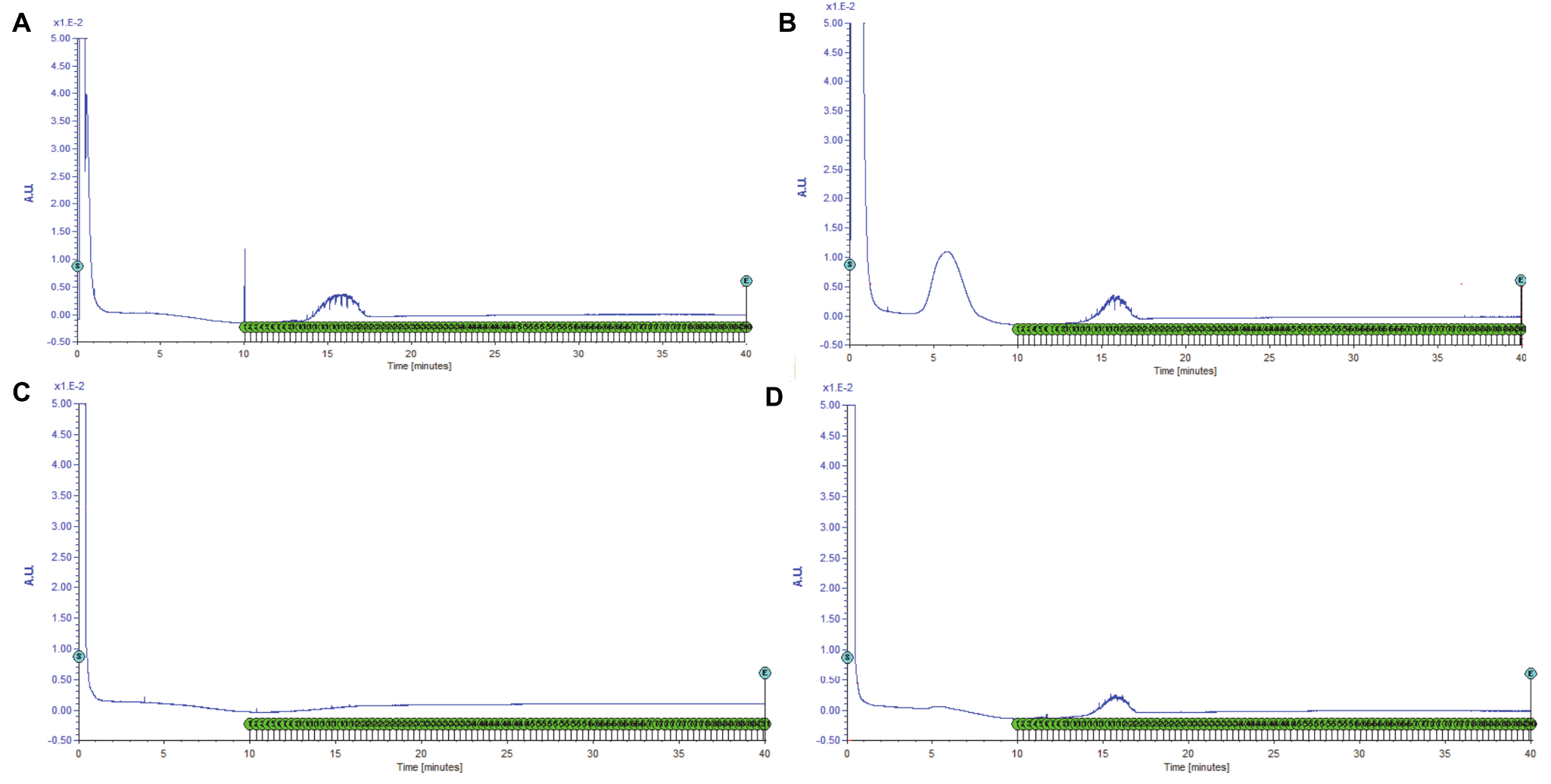

Supplement: Figure S2 — Chromatograms of selection rounds. A) Round 1, drug disk; B) Round 1, blank disk; C) Round 2, drug disk; D) Round 2, blank disk. The green circles correspond to individual fractions collected. (TIF) [file pone.0057341.s002.tif]

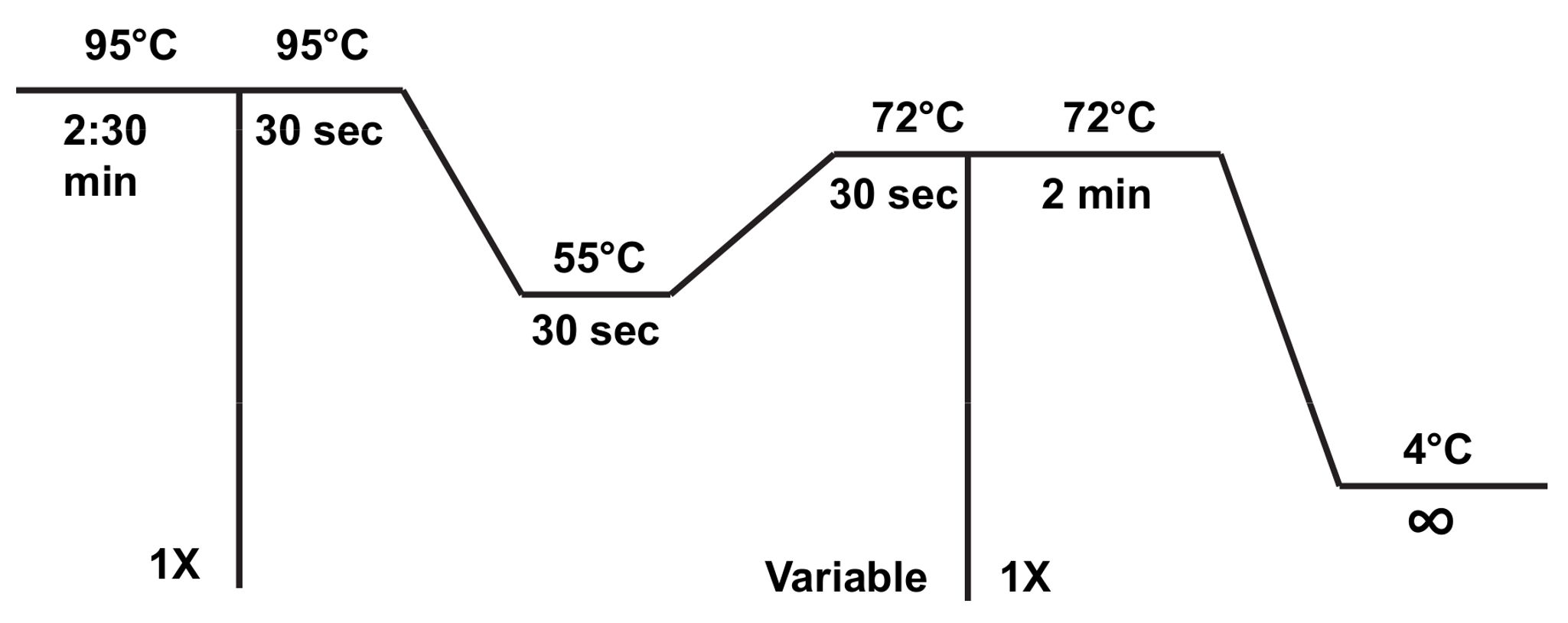

Supplement: Figure S3 — PCR protocol for amplification. (TIF) [file pone.0057341.s003.tif]

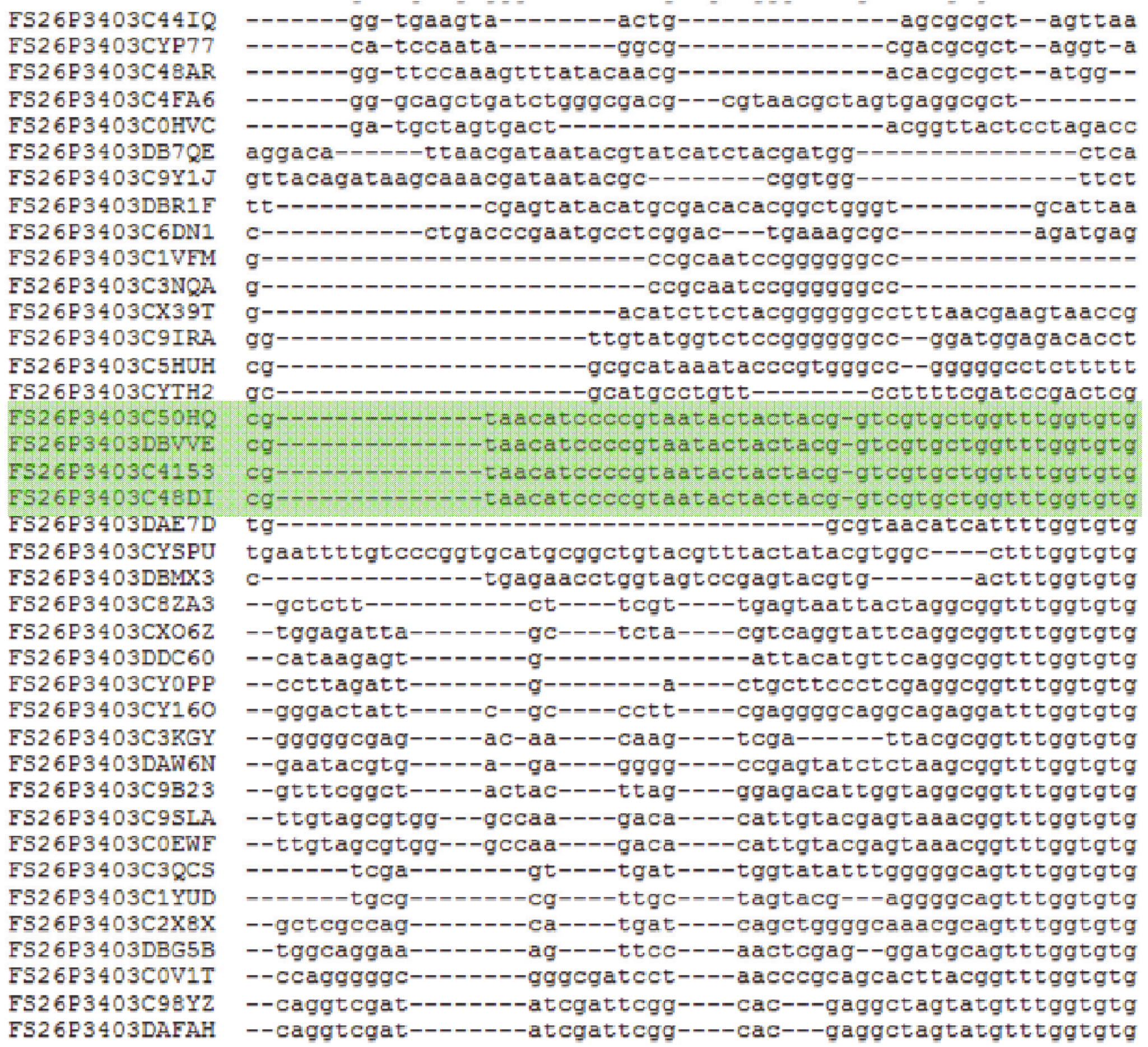

Supplement: Figure S4 — Sample alignment of JPB2 (green highlighted portion) using MAFFT. (TIF) [file pone.0057341.s004.tif]

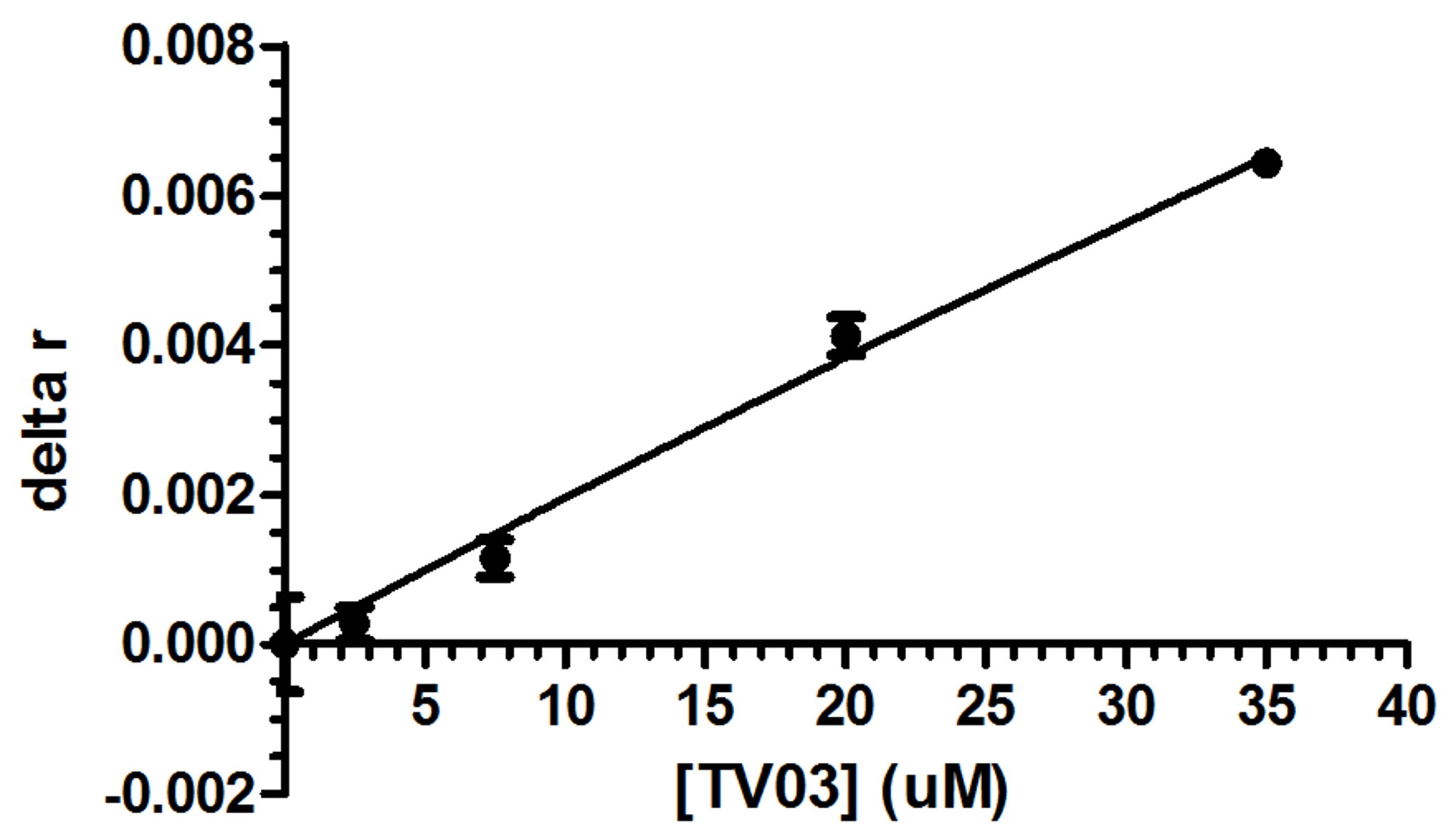

Supplement: Figure S5 — Dissociation constant curve of control sequence TV03. (TIF) [file pone.0057341.s005.tif]

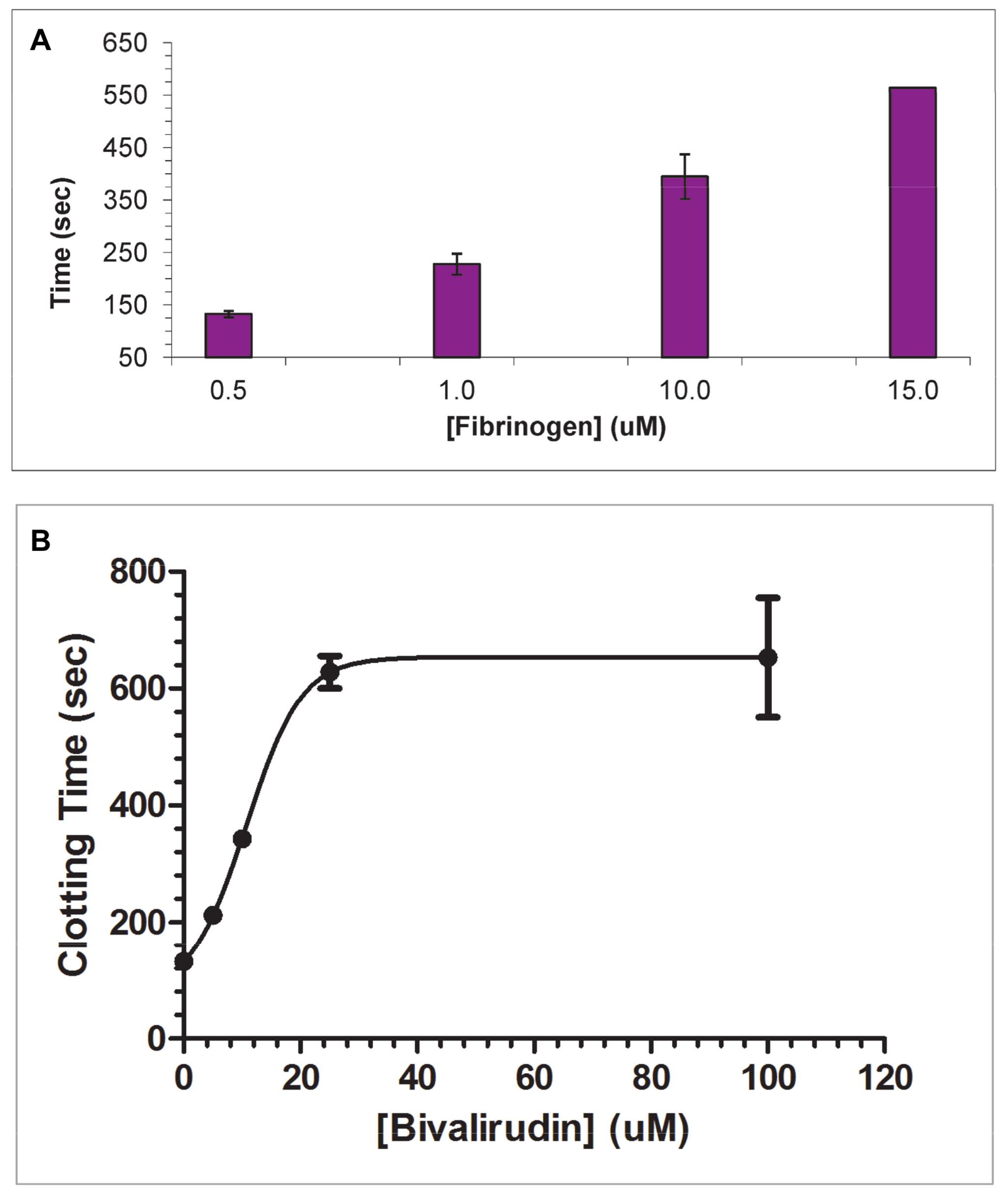

Supplement: Figure S6 — Optimization of conditions for buffer clotting experiments. A) Optimization of fibrinogen concentration; B) Optimization of bivalirudin concentration. (TIF) [file pone.0057341.s006.tif]

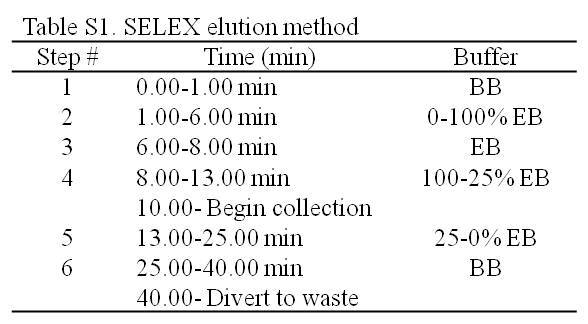

Supplement: Table S1 — SELEX DNA elution method (JPG) [file pone.0057341.s007.jpg]

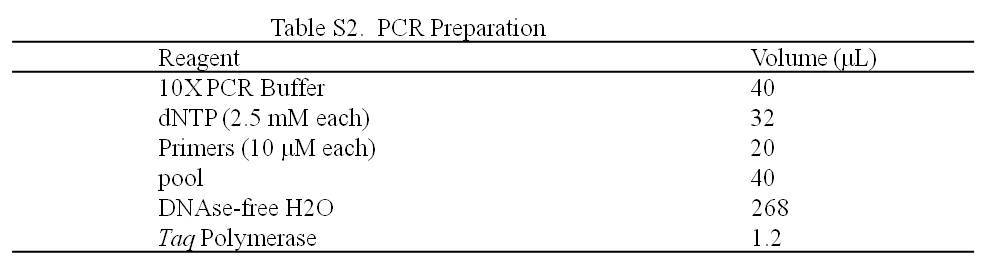

Supplement: Table S2 — PCR Preparation (JPG) [file pone.0057341.s008.jpg]

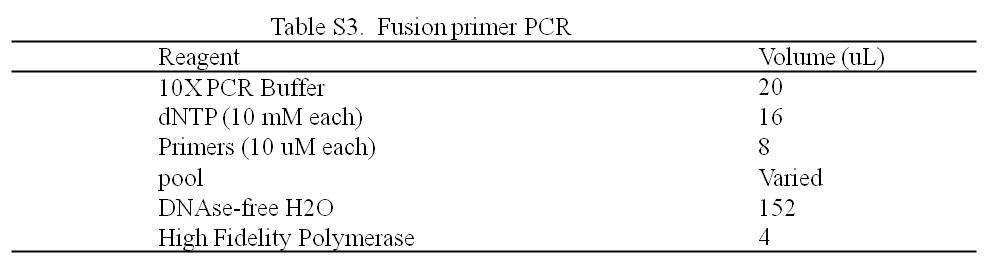

Supplement: Table S3 — Fusion primer PCR (JPG) [file pone.0057341.s009.jpg]
