# Supplementary material for: Selection of an Aptamer Antidote to the Anticoagulant Drug Bivalirudin
Source: PLoS One. 2013 Mar 6;8(3):e57341. doi: 10.1371/journal.pone.0057341 (PMC3590194; doi:10.1371/journal.pone.0057341)
Supplement: Text S1 — Supporting Information (DOCX) [file pone.0057341.s010.docx]

**Selection of an Aptamer Antidote to the Anticoagulant Drug Bivalirudin**

Jennifer A. Martin^1^, Parag Parekh^1^, Youngmi Kim^1^, Timothy E. Morey^2^, Kwame Sefah^1^, Nikolaus Gravenstein^2^, Donn M. Dennis^2^ and Weihong Tan^1^

1. Department of Chemistry and Department of Physiology and Functional Genomics, Shands Cancer Center and Center for Research at the Bio/nano Interface, University of Florida, Gainesville, FL 32611-7200

2. Department of Anesthesiology and Pharmacology, University of Florida, Gainesville, FL 32611-7200

CORRESPONDING AUTHOR: Weihong Tan, Department of Chemistry and Shands Cancer Center, University of Florida, Gainesville, FL 32611; Tel and Fax: 352-846-2410; e-mail: [tan@chem.ufl.edu.](mailto:tan@chem.ufl.edu.)

**Supporting Information**

**DNA Synthesis.** All DNA synthesis reagents were purchased from Glen Research. The sequences were synthesized using an ABI3400 DNA/RNA synthesizer (Applied Biosystems) at the 1 μmol scale. For cleavage and deprotection, incubation with ammonium hydroxide was followed by ethanol precipitation. The precipitates were dissolved in 0.5 mL of 0.1 M triethylammonium acetate (TEAA, pH=7.0), and DNA purification was performed with a ProStar HPLC (Varian) using a C18 column (Econosil, 5U, 250 mm × 4.6 mm) from Alltech Associates. UV-Vis measurements were performed with a Cary Bio-300 UV spectrometer (Varian) to measure DNA concentration.

**Peptide Immobilization.** Buffers- binding buffer (BB): PBS buffer (no MgCl_2_ or CaCl_2_) with 5 mM MgCl_2_ (final concentration); elution buffer (EB): BB with 1 M NaCl. All buffers and reagents added to the monolithic column were first filtered with a 0.22 μm cellulose nitrate filter (Corning). Bivalirudin (Angiomax; The Medicines Company) was received in lyophilized form as a gift from the Anesthesiology Department at the University of Florida College of Medicine. The peptide was immobilized on an epoxy-functionalized CIM disk (BIA Separations) following manufacturer’s instructions (Figure S1). Briefly, the column was inserted into the disk housing and equilibrated with 2 mL of BB at 1 mL/min, then 2 mL of EB at 1 mL/min, and finally 2 mL BB at 2 mL/min using a low-pressure chromatography device (LPC; Bio-Rad BioLogic LP). Drug immobilization began with a 2 mL wash with 0.5 M sodium phosphate buffer (pH 8.0) at a flow rate of 2 mL/min. The drug was dissolved in 5 mL of sodium phosphate buffer (pH 8.0) for a final concentration of 3.0 mg/mL. This solution was introduced onto the disk (1 mL), and the disk was covered and incubated with the eluate for 48 hours at room temperature in a small dish. For the blank disk (no drug immobilized), the same buffer without drug was incubated with the disk, and the remainder of the protocol was followed. Following this step, the disk was washed with 2 mL 0.5 M sodium phosphate buffer (pH 8.0), and remaining epoxy groups were blocked by flowing 4 mL of 1 M ethanolamine through the disk and incubating overnight at room temperature in a dish. The reaction was quenched by washing the disk with 2 mL of 0.5 M sodium phosphate buffer (pH 8.0) with 1 M NaCl at 2 mL/min and equilibrated for use by introducing 2 mL of 0.5 M sodium phosphate buffer (pH 8.0) at 2 mL/min. The column was washed in 2 mL BB and sealed in the column housing with the column blind fittings for storage at 4°C for usage planned less than 3 days after last usage, or washed with 4 mL of 20% ethanol and stored at 4°C in a container with 20% ethanol if the next usage was planned for longer than three days.

**Quantitation of Peptide Immobilization.** The peptide solution remaining after the 48-hour incubation with the disk was collected and analyzed by UV/Vis (Bio-Rad SmartSpec Plus) at λ_abs_=280 nm. The mass of peptide remaining in the solution was calculated and subtracted from the initial amount added for the immobilization to determine that 1 mg of peptide was immobilized on the disk.

**Library and Primers.** The DNA library of sequence 5'-ATC GTC TGC TCC GTC CAA TA -N_46_- TTT GGT GTG AGG TCG TGC and primer sequence 5’-ATC GTC TGC TCC GTC CAA TA were synthesized and purified as described above. Primer sequence 5'-Biotin-GCA CGA CCT CAC ACC AAA was purchased from IDT.

**SELEX Methods.** The first round of selection generated the chromatograms seen in Figure S2A-B. The peak at 15 min for the drug disk appeared to have a larger area than that of the blank disk. Eluate corresponding to the entire peak observed in the drug disk chromatogram was combined and incubated with the blank disk to subtract out sequences binding to the column matrix. The DNA pool remaining after the counter- selection was PCR-amplified and prepared for the second round of selection.

In the second round, a chromatographic difference between the disks was also observed (Figure S1C-D), but this time the drug disk did not display a noticeable peak. This may have been caused by the decrease in the amount of DNA used from the first round (1 nmol) to the second round (0.2 nmol), resulting in a signal below the limit of detection of the system. A similar selection using (0.2 nmol) DNA was also performed on the blank; however, a small peak was observed at 15 min for the blank disk. Groups of fractions were combined and prepared for qPCR analysis, corresponding to fractions 80-89 and 70-79 from the drug and blank selections respectively.

| Table S1. SELEX DNA elution method | | |
| --- | --- | --- |
| Step # | Time (min) | Buffer |
| 1 | 0.00-1.00 min | BB |
| 2 | 1.00-6.00 min | 0-100% EB |
| 3 | 6.00-8.00 min | EB |
| 4 | 8.00-13.00 min | 100-25% EB |
|  | 10.00- Begin collection |  |
| 5 | 13.00-25.00 min | 25-0% EB |
| 6 | 25.00-40.00 min | BB |
|  | 40.00- Divert to waste |  |

**454 Sequencing Preparation.** A 400 μL (total volume) 25 cycle PCR (Table S2) was performed on the selected pool according to the protocol in Figure S3. This dsDNA-amplified pool was prepared for sequencing by high-fidelity (Roche Applied Science FastStart High Fidelity PCR System) PCR amplification with fusion primers specific for 454 sequencing technology. The primer sequences used for PCR were composed of a combination of the 454 fusion tag and the regular sense and anti-sense primers previously used for amplification:

Sense: GCC TCC CTC GCG CCA TCA GAT CGT CTG CTC CGT CCA ATA

Anti-sense: GCC TCC CTC GCG CCA TCA GAT CGT CTG CTC CGT CCA ATA

| Table S2. PCR Preparation | |
| --- | --- |
| Reagent | Volume (μL) |
| 10X PCR Buffer | 40 |
| dNTP (2.5 mM each) | 32 |
| Primers (10 μM each) | 20 |
| DNA pool | 40 |
| DNAse-free H2O | 268 |
| *Taq* Polymerase | 1.2 |

The fusion primer PCR also utilized the protocol from Figure S3, but it was prepared by adding 0.5 μL of the dsDNA to 50 μL of the PCR reaction mixture prepared from Table S3. UV spectroscopy was used to calculate the concentration of DNA produced as 452 μg/mL.

| Table S3. Fusion primer PCR | |
| --- | --- |
| Reagent | Volume (uL) |
| 10X PCR Buffer | 20 |
| dNTP (10 mM each) | 16 |
| Primers (10 uM each) | 8 |
| DNA pool | Varied |
| DNAse-free H2O | 152 |
| High Fidelity Polymerase | 4 |

The dsDNA product was purified by a QiaQuick PCR Purification Kit (Qiagen) following the manufacturer’s instructions. Buffer PB (125 μL; Qiagen proprietary buffer) was added to the PCR product, and the sample was centrifuged at 13,000 rpm for 45 sec in a QiaQuick column. The eluate was discarded, and 0.75 mL Buffer PE (Qiagen buffer) was added to the column and centrifuged for 1 min at 13,000 rpm. This eluate was discarded as well, and the remaining product was centrifuged 1 min at 13,000 rpm. The filter portion was placed in a clean 1.5 mL microcentrifuge tube, and the purified product was eluted with 50 μL Qiagen buffer EB by centrifugation (13,000 rpm, 1 min). The concentration was determined by UV spectroscopy to be 24 μg/mL, which was diluted to 4.2 μg/mL with the Qiagen EB buffer according to 454 procedures (request samples ~5 μg/mL). Sequencing of the selected pool was performed by 454 sequencing at the University of Florida ICBR.

**Sequence Alignment.** Sequences obtained from 454 sequencing were aligned using MAFFT. An example is shown in Figure S4.

**Drug Biotinylation and Purification for Binding Assay.** The drug was biotinylated by combining 41.9 mg of Angiomax and 6.2 mg of biotin (EZ-Link Sulfo-NHS-SS-Biotin; Pierce) with 500 μL of PBS buffer (no MgCl_2_ or CaCl_2_ added) and stirring with a magnetic stir bar and plate overnight at 4°C. HPLC purification, MALDI analysis (Applied Biosystems 4700 Proteomics Analyzer), and lyophilization were performed by the University of Florida ICBR.

**Fluorescence Anisotropy for Dissociation Constant (K_d_) Measurements.** The drug was conjugated to 5,6-NHS-TMR (5,6-N-hydroxysuccinimide-carboxytetramethylrhodamine; AnaSpec) in a manner similar to that described for the drug/biotin conjugation. In this case, 5 mg of TMR dye was dissolved in PBS buffer (no MgCl_2_ or CaCl_2_ added) and DMSO and slowly added to 46.5 mg Angiomax in 100 μL PBS buffer for a total volume of 600 μL. The solution was stirred overnight at 4°C and HPLC purified as described for the biotin/drug conjugation. Anisotropy measurements were performed on a Fluoromax-4 spectrofluorometer (Horiba Jobin Yvon) with excitation and emission polarizers in L-format. Based on the manufacturer’s recommendations, the following settings were determined: λ_Ex_= 545 nm; λ_Em_= 580 nm; integration time= 2 sec; slit width= 7 nm (JPB2) or 9 nm (JPB5, TV03); peptide concentration= 1 μM; total volume= 200 μL. DNA concentrations in the micromolar range were titrated into the peptide solution and incubated for 1 min. A minimum of four measurements were taken for each aptamer concentration. The results were plotted in GraphPad Prism as change in anisotropy versus aptamer concentration using a single-site ligand binding model to determine the K_d_. The change in anisotropy was the average anisotropy of the initial dye-labeled peptide subtracted from the average anisotropy value at each aptamer concentration. As a control, the curve generated by aptamer TV03, which binds virus infected cells (1), was also generated with bivalirudin as the target (Figure S5).

**Optimization of Buffer Clotting Experiment Conditions.** The concentration of fibrinogen used in the assay was optimized based on a physiologically relevant concentration of thrombin. In this experiment, the reaction was carried out in a 100 μL quartz fluorescence cuvette (Starna Cells), and the light scattering was monitored on a Fluoromax-4 spectrofluorometer (Horiba Jobin Yvon). The settings for this experiment were as follows: λ= 500 nm; slit width= 3 nm; integration time= 0.1 sec; interval= 0.5 sec, temperature= 37°C. The normal clotting time was determined by adding 50 μL of 15.5 nM thrombin (human α-thrombin; Haematologic Technologies) in BB to the cuvette and equilibrating it in the instrument for 2 minutes (step 1). Next, 50 μL of fibrinogen (Sigma-Aldrich) concentrations ranging from 0.5-15 μM (final) were added and mixed well by pipette (step 2), and the increase in light scattering was monitored using FluorEssence software. Each fibrinogen concentration was tested twice (with the exception of 15 μM fibrinogen), and each measurement of light intensity versus time was best-fit to a 4-parameter sigmoid equation using GraphPad Prism to determine the clotting time.

The clotting time was observed to decrease as the fibrinogen concentration decreased (Figure S6), which is counterintuitive to expected results, as a decrease in substrate is generally believed to result in a decrease in product formation. Product inhibition may provide one explanation for this phenomenon. Since most coagulation factors have negative feedback activity, product inhibition becomes especially relevant. That is, the products can inhibit upstream reactants once the concentration exceeds normal limits (2). This function balances the processes so the body remains in homeostasis. Fibrin strands are known to retain weak binding to thrombin, so it is possible that the excess fibrin product at exosite 1 inhibits binding of fibrinogen to the same site (3). Also, the lack of all constituents of the coagulation cascade changes the dynamics of the system, where the different components may stabilize this effect.

Building the dose response curve commenced using the same settings as those used for the fibrinogen concentration experiments. In these experiments, 50 μL of 15.5 nM thrombin in BB and drug concentrations ranging from 0-100 nM final concentration were added to the cuvette and equilibrated in the instrument for 2 minutes (step 1). Next, 50 μL of 0.5 μM (final) fibrinogen was added and mixed well by pipette (step 2), and the increase in light scattering was monitored using FluorEssence software. Each drug concentration was tested twice; then each curve of light intensity versus time was best-fit to a 4-parameter sigmoid equation using GraphPad Prism to determine the clotting time (considered as the halfway point between the minimum and maximum intensity values). These coagulation times at each drug concentration were then averaged, plotted versus the drug concentration, and best-fit to the same 4-parameter sigmoid equation. The drug concentration required to double the normal clotting time was considered to be the concentration at the halfway point between the minimum and maximum clotting time values.

1. Tang, Z. W., Parekh, P., Turner, P., Moyer, R. W., & Tan, W. H. (2009) *Clinical chemistry* **55,** 813-822.

2. Holmes, V. A. & Wallace, J. M. W. (2005) *Biochem. Soc. Trans.* **33,** 428-432.

3. Weitz, J. I., Hudoba, M., Massel, D., Maraganore, J., & Hirsh, J. (1990) *J. Clin. Invest.* **86,** 385-391.

**Supporting Information Legends**

**Figure S1. Immobilization scheme of bivalirudin to the monolithic column.**

**Figure S2.** **Chromatograms of selection rounds.** A) Round 1, drug disk; B) Round 1, blank disk; C) Round 2, drug disk; D) Round 2, blank disk. The green circles correspond to individual fractions collected.

**Figure S3.** **PCR protocol for amplification.**

**Figure S4.** **Sample alignment of JPB2 (green highlighted portion) using MAFFT.**

**Figure S5.** **Dissociation constant curve of control sequence TV03.**

**Figure S6.** **Optimization of conditions for buffer clotting experiments.** A) Optimization of fibrinogen concentration; B) Optimization of bivalirudin concentration.

**Table S1. SELEX DNA elution method**

**Table S2. PCR Preparation**

**Table S3. Fusion primer PCR**
